# Transcranial Direct Current Stimulation (tDCS) in Unilateral Cerebral Palsy: A Pilot Study of Motor Effect

**DOI:** 10.1155/2019/2184398

**Published:** 2019-01-14

**Authors:** Emanuela Inguaggiato, Nadia Bolognini, Simona Fiori, Giovanni Cioni

**Affiliations:** ^1^Department of Developmental Neuroscience, IRCCS Fondazione Stella Maris, Calambrone, Pisa, Italy; ^2^Department of Psychology, NeuroMi, University of Milano Bicocca, Milan, Italy; ^3^Laboratory of Neuropsychology, IRCCS Istituto Auxologico Italiano, Milan, Italy; ^4^Department of Clinical and Experimental Medicine, University of Pisa, Pisa, Italy

## Abstract

Transcranial Direct Current Stimulation (tDCS) is an emerging tool to improve upper limb motor functions after stroke acquired in adulthood; however, there is a paucity of reports on its efficacy for upper limb motor rehabilitation in congenital or early-acquired stroke. In this pilot study we have explored, for the first time, the immediate effects, and their short-term persistence, of a single application of anodal tDCS on chronic upper limb motor disorders in children and young individuals with Unilateral Cerebral Palsy (UCP). To this aim, in a crossover sham-controlled study, eight subjects aged 10-28 years with UCP underwent two sessions of *active* and *sham* tDCS. Anodal tDCS (1.5 mA, 20 min) was delivered over the primary motor cortex (M1) of the ipsilesional hemisphere. Results showed, only following the active stimulation, an immediate improvement in unimanual gross motor dexterity of hemiplegic, but not of nonhemiplegic, hand in Box and Block test (BBT). Such improvement remained stable for at least 90 minutes. Performance of both hands in Hand Grip Strength test was not modified by anodal tDCS. Improvement in BBT was unrelated to participants' age or lesion size, as revealed by MRI data analysis. No serious adverse effects occurred after tDCS; some mild and transient side effects (e.g., headache, tingling, and itchiness) were reported in a limited number of cases. This study provides an innovative contribution to scientific literature on the efficacy and safety of anodal tDCS in UCP. This trial is registered with NCT03137940.

## 1. Introduction

Unilateral Cerebral Palsy (UCP) represents the most frequent form of CP, affecting about 30%-40% of all children with CP [1]. In general, the upper limb is more involved, impacting daily use of hand in activities such as reaching, grasping, and manipulation of objects. UCP is associated with heterogeneous brain lesions, mainly due to perinatal stroke, and its clinical manifestation is related to timing (acquired vs. congenital, acute vs. chronic) and etiology of brain injury [1]. The hand contralateral to the nondamaged or less damaged hemisphere may be underperforming, compared to typically developing children, and therefore, the terms more-affected and less-affected hand, instead of affected and nonaffected, have been suggested in studies with children with UCP [2]. In order to improve functions of the hemiplegic hand, several types of intervention have been used with some success. Recently, there has been an increasing interest in the use of Noninvasive Brain Stimulation (NIBS) techniques, such as transcranial magnetic (TMS) and direct current electrical stimulation (tDCS), to enhance poststroke motor disorders and neurodevelopmental outcomes.

With tDCS, continuous and weak electric currents (typically 0.5-2.0 mA) are applied over the scalp in order to modulate brain activity [3–5]. On the neuronal level, the primary mechanism of action is a polarity-dependent shift (polarization) of the resting membrane potential. While anodal stimulation generally enhances motor cortical excitability, cathodal stimulation has the opposite effect, decreasing cortical excitability. This polarization mechanism underlies the acute, short-lasting, and reversible effects of tDCS in humans [6]. Multiple consecutive applications of tDCS are required to induce persistent after-effects with cortical excitability shifts maintained in the long term. Such after-effects involve modification of synaptic microenvironment and are mediated by GABA and NMDA receptors, which subtend synaptic plasticity mechanisms similar to those observed in long-term potentiation (LTP) and depression (LDP) [6, 7]. It is important to remember that the excitatory and inhibitory effects of anodal and cathodal stimulations depend on various factors, most of which are still unknown. Indeed, a growing body of evidence shows that tDCS does not function in a linear manner, so that physiological and behavioral outcomes, in terms of facilitation or inhibition of cortical excitability, depend on the interaction of several factors related not only to technical parameters, such as current polarity and duration, but also to individual and task characteristics, as well as to metaplasticity-related effects [8]. This is especially relevant, and even more complex, in a developing brain [9].

The fact that plasticity-dependent after-effects induced by tDCS are associated with long-term behavioral improvements has fostered clinical research on the therapeutic potential of this technique for the treatment of neurological and psychiatric diseases [10]. With respect to rehabilitation of poststroke motor disorders in adults, two main approaches have been tested in line with a model proposing the existence of a maladaptive interhemispheric imbalance between the two hemispheres after a unilateral stroke [11]. Following this model, poststroke motor recovery may be facilitated by either upregulating the excitability of the lesioned motor cortex (through anodal tDCS) or by downregulating the hyperexcitability of the intact motor cortex (through cathodal tDCS) [12–14]. Though the principal theory differs in many aspects, these two approaches have been adopted also for improving upper limb motor disorders in subjects with UCP. Indeed, current knowledge recognizes, as main components of developmental neuroplasticity following perinatal brain injury, both influences of contralateral and ipsilateral corticospinal projections to the paretic hand, and the intrahemispheric and interhemispheric connections of the lesioned and intact motor cortices. It follows that the damaged as well as the intact motor cortex may represent potential central therapeutic targets for tDCS in UCP [9, 15, 16]. It is also important to consider that this neuromodulation tool can modulate the activity and functional connectivity of large-scale brain networks in both hemispheres, even when the stimulating electrode is applied “unilaterally,” over a specific cortical region, such as M1 [15].

In the pediatric population, there is a paucity of research on the therapeutic potential of tDCS, with respect to both clinical efficacy and safety in children and young individuals with neurodevelopmental disorders. Some limited data exist from research conducted on ADHD, autism, epilepsy, and learning disorders [17]; they confirm the feasibility and safety of tDCS in the pediatric population, describing some positive clinical effects obtained in the treatment of these disorders. In individuals with CP, single or multiple applications of anodal tDCS over the primary motor cortex (M1) of the affected, or more affected, hemisphere seem to improve gait and reduce muscle spasticity [18, 19]. An essential central concept that has emerged from therapeutic brain stimulation studies in adults is the need to stimulate motor learning in the injured brain. To facilitate motor recovery, tDCS should be used as an add-on intervention to motor therapies in clinical settings [20–24]. In this regard, some promising effects on manual functions have been obtained applying, during a motor therapy, cathodal tDCS over the intact hemisphere [25, 26]. However, tDCS efficacy for driving upper limb motor recovery in UCP still requires further research.

In this context, the main aim of our pilot, proof-of-principle, study was to evaluate, for the first time, the effect of a single anodal tDCS application over the ipsilesional motor cortex on the unilateral gross manual function of the more affected, hemiplegic, contralesional hand in a small group of subjects with UCP, while also exploring the possible influences of demographic and lesion factors. We measured both the immediate effects of tDCS (i.e., acute effects emerging immediately at the end of the stimulation) and their persistence in the short-term (within 90 minutes poststimulation). We focused on short-term effects since seminal neurophysiological studies in humans have showed that a single application of anodal tDCS for 13 minutes can induce an increase of motor cortex excitability (as indexed by increased amplitude of motor-evoked potentials induced by single-pulse TMS) that persists for a maximum duration of 1.5 hours after stimulation [27]. We adopted a study design similar to that used in stroke adults in the original study by Boggio and colleagues [28], who investigated the possible modulation induced by a single application of tDCS on the motor functions of the paretic hand in stroke adults. As in Boggio's study, this study did not combine tDCS with motor learning tasks. We also assessed tDCS effects on the motor function of the nonhemiplegic hand, its safety and tolerability by monitoring possible side effects and effect on blood pressure and heart rate.

Anodal tDCS was applied for 20 minutes, with an intensity of 1.5 mA. These tDCS parameters (intensity and duration) were chosen in light of previous evidence in stroke adults [6, 8–13, 19]. Both in children with typical development and in children with UCP, current evidence is still insufficient to delineate the optimal tDCS dosage (i.e., current intensity and duration) for modulating motor performance. In studies investigating tDCS effects in pediatric populations, current intensities have ranged from 0.3 to 2.0 mA (most frequently 1 mA), with a duration up to 20 minutes [29]. In children with UCP, only cathodal stimulation, administered as adjuvant to motor therapy, has been used to modulate upper limb motor functions. In this case, it was shown that an intensity equal to or below 1 mA was unable to increase gains of motor training, as compared to the add-on use of sham tDCS (at least with respect to objective motor outcomes) [25, 26, 30]. In a study on healthy children assessing tDCS effects on motor learning, cathodal stimulation at 2 mA was shown to be less effective than anodal and cathodal stimulations at 1 mA [30]. So far, a current intensity of 1.5 mA was never tested in UCP [29], while there is evidence of its efficacy in adult stroke (e.g., [19]).

## 2. Materials and Methods

### 2.1. Participants

**Figure 1:**
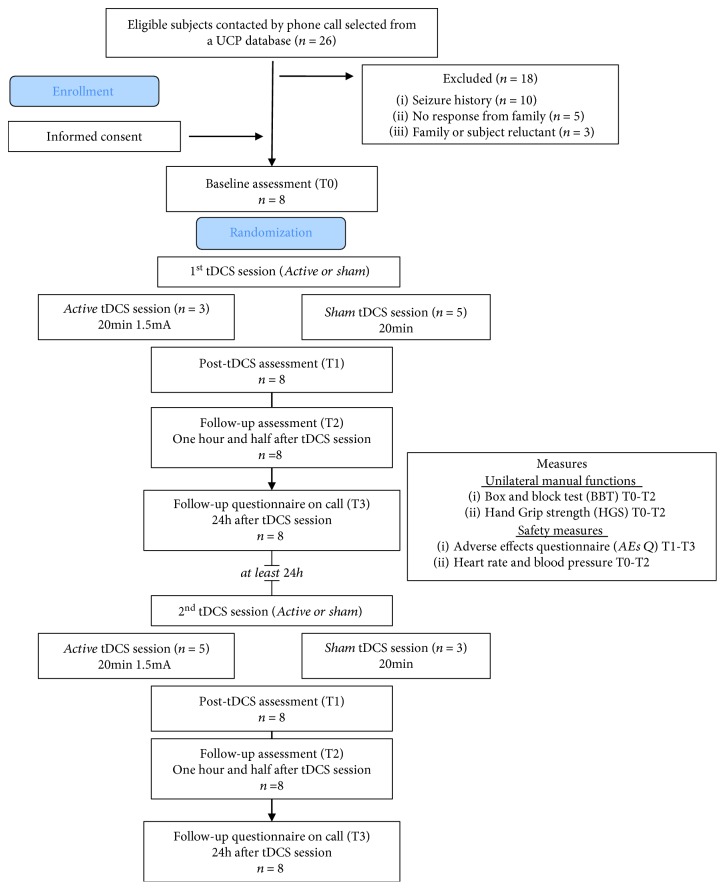
CONSORT diagram and study flow.

Participants were selected from the UCP database of the IRCCS Stella Maris Foundation (Pisa, IT), according to the following inclusion criteria: (1) diagnosis of UCP, confirmed by brain MRI indicating congenital unilateral brain lesion (i.e., a lesion that occurs either prenatally or perinatally within 28 days from birth), (2) aged between 10 and 28 years, (3) absence of history of seizures or epilepsy, and (4) no contraindication to tDCS [31–33]. Subjects were excluded if one of the following conditions exists: (1) epilepsy or first degree relative with epilepsy (in some cases the presence of epilepsy was identified after selection from database and therefore subsequently excluded) [33], (2) bilateral lesion, (3) other severe concomitant disabilities, and (4) botulinum toxin for the upper limb within the last 6 months. Contacted participants were also selected on the basis of residence: we excluded subjects that lived more than 100 km from IRCCS Stella Maris Foundation. After section and telephone contact to verify eligibility and potential interest of subjects and their families for the study, eight participants (mean age = 17.5 ± 6.1, range = 10-22 years) were recruited (Figure 1).

Functional hand level was determined according to the Manual Ability Classification System (MACS, Italian translation, 2010) [34]. Clinical and demographic features of participants are reported in Table 1.

This study was conducted according to the Good Clinical Practice and was approved by the Tuscan Region Pediatric Ethics Committee (Florence, Italy) in March 2016. The study began in June 2016 and finished in October 2017. The trial has been registered in the ClinicalTrials.gov (ClinicalTrials.gov Identifier: NCT03137940). Participants were informed that they could voluntarily withdraw from the study at any time.

### 2.2. Structural MRI

Each participant underwent structural MRIs, on which severity of lesion was classified using a qualitative classification related to timing of brain insult in UCP [35] and a semiquantitative scale for brain lesion severity by a pediatric neurologist (SF), with expertise in neuroimaging [36, 37]. Timing of insult results in three forms of congenital brain lesions [35], corresponding to brain maldevelopments (first two trimesters of pregnancy), periventricular venous infarction (early third trimester), and ischemic stroke (later third trimester). The semiquantitative scale described by Fiori and colleagues [36, 37] is a reliable system for the classification of brain lesion severity in children with CP. According to this scale, brain lesions are represented on a graphical template and raw scores for each region of the brain are systematically calculated, where higher scores represent more severe pathologies (i.e., a larger lesion within a given region as indicated by signal change and missing tissue). Hemispheric score is the sum of lobar scores (maximum score of 12) in each hemisphere. Basal ganglia and brainstem score is the sum of subcortical structures (basal ganglia, thalamus, brainstem, and posterior limb of internal capsule: maximum score of 5) on each side, and the global score is the sum of the right and left hemispheric scores, basal ganglia and brainstem scores, and corpus callosum and cerebellum scores (maximum score of 40).

### 2.3. Transcranial Direct Current Stimulation: tDCS

tDCS was delivered by a battery-powered constant current stimulator (BrainStim, E.M.S. s.r.l., Bologna, Italy; http://brainstim.it) using a pair of surface saline-soaked sponge electrodes placed on the scalp. The anodal electrode was placed over C3 or C4 (according to the 10-20 electroencephalograph system for electrode placement) in order to stimulate the primary motor cortex (M1) of the damaged hemisphere, with the cathode electrode placed over the contralateral supraorbital area. During active tDCS, a constant current of 1.5 mA was applied for 20 minutes, with a ramping period of 30 seconds at both the beginning and end of stimulation (i.e., fade-in and fade-out phases, respectively).

Sham tDCS was applied with the same parameters and electrode montage as active tDCS, but the current lasted only 30 seconds [38]. Sham and active modes of the tDCS device were set in advance by one of the investigators (NB), who did not participate in data collection, thus keeping both participant and investigator applying tDCS and collecting data blind. This sham procedure is commonly used in clinical investigation [5].

### 2.4. Outcome Measures

#### 2.4.1. Box and Block Test (BBT)

BBT is a highly reliable hand dexterity test [39, 40], composed of a box and divided into two compartments, containing 150 wooden cubes (2.5 cm^3^). Participants are instructed to grasp a wooden cube from one side of the box and drop it into the opposite side. Subjects perform a 1-minute trial, grasping and releasing as many blocks as possible and performance is measured by the number of blocks transferred in 1 min. If the subject transfers two or more cubes at the same time, this number is subtracted from the total score. According to BBT instructions, a 15-second practice preceded testing. The test was video-recorded for off-line analyses.

#### 2.4.2. Hand Grip Strength (HGS) Test

The HGS measures (in kg) the maximum voluntary isometric strength of the hand, through a hydraulic hand dynamometer (the mean of three trials was taken as score).

### 2.5. Safety Questionnaire, Blood Pressure, and Heart Rate

A questionnaire, adapted from Bolognini et al. [24, 41, 42], was used to monitor adverse effects of tDCS; these items are illustrated in Tables 2 and 3 and examined the occurrence of the most common tDCS side effects (e.g., itchiness, headache, and tingling) [31]. Adaptation of the questionnaire consisted in the substitution of some specific terms to make it easier for children to understand; moreover, a specific section for follow-up assessment after 24 hours was inserted to assess day-after changes in mood, daily activities, and quality of sleep.

If an adverse effect was reported, the participant had to rate its intensity (0 = absent, 1 = mild, 2 = moderate, and 3 = severe) and report whether, in their view, the reported sensation was related or not to tDCS (0 = no correlation, 1 = possible, 2 = probably, and 4 = surely). Moreover, at the end of questionnaire, the experimenter also inquired, in an informal way, about the overall well-being and general feeling of participants and their caregivers with reference to tDCS.

Blood pressure and heart rate were evaluated using an automatic device (Boso medicus machine; Bosch+Sohn GMBH, Germany).

### 2.6. Experimental Design

We adopted a crossover, double-blind, sham-controlled design, with all participants undergoing two tDCS sessions, one with active and one with sham tDCS (in a random order across participants). In both sessions, motor functions (BBT, HGS), heart rate, and blood pressure were assessed immediately before tDCS (T0, i.e., baseline), immediately after (T1), and 90 minutes after the end of tDCS (T2) (Figure 1).

The tDCS questionnaire was administered at the end of each tDCS session (T1 and T2; Table 2) and the day after, through a phone call (T3; see Table 3). Since this was a pilot study, with explorative purposes, a sample-size calculation was not performed.

### 2.7. Statistical Analyses

Statistical analyses were carried out with SPSS (IBM SPSS Statistic version 21).

Considering that each participant underwent two stimulation sessions (active and sham tDCS) and that the evaluations were performed at 3 time points (baseline, T0, immediately and 90 min after tDCS, T1 and T2, respectively), a repeated-measure analysis of variance (rmANOVA) was used to evaluate the effects of within-factor tDCS (active, sham) and Time (T0, T1, and T2) on BBT and HGS scores, separately for the hemiplegic and nonhemiplegic hands. We separately analyzed the two hands since modulation of motor performance of the hemiplegic hand represented our primary outcome. Moreover, we recognized the exploratory nature of this study on a small sample. Effects were also evaluated according to a standardized size-effect index that is partial eta-squared (p*η*^2^). For significant effects, post-hoc testing was performed and corrected for multiple comparisons (Bonferroni). In every analysis, the significance level was set at *p* < 0.05. All data are expressed as mean ± SE.

Preliminary testing for normality with the Shapiro-Wilk test showed that, in every test (BBT, HGS, blood pressure, and heart rate), data were normally distributed (all *p* > 0.09) in all assessments (T0-T1-T2, of both active and sham tDCS sessions). Moreover, before running the analyses, the sphericity requirements for rmANOVAs were assessed by using Mauchly's test; whenever assumptions were not met, Greenhouse-Geisser correction was used for violations of sphericity.

## 3. Results

### 3.1. Box and Block Test and Hand Grip Strength

**Figure 2:**
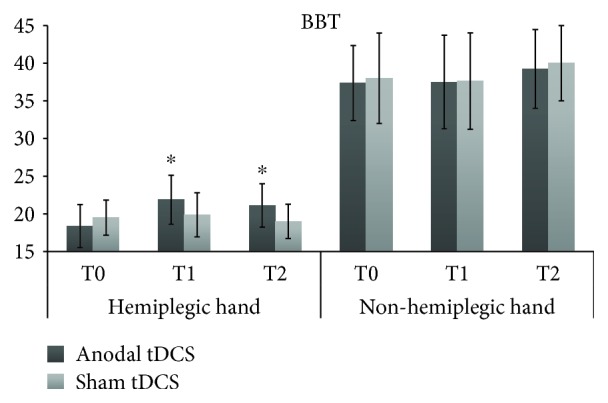
BBT scores (i.e., number of blocks moved in 1 min) for the hemiplegic and nonhemiplegic hands, at each assessment of the active anodal tDCS and sham tDCS sessions. T0 = baseline; T1 = immediately after the end of tDCS; T2 = 90 min after the stimulation session. ^∗^ = significant change from baseline, *p* < 0.05.

With respect to the performance of the hemiplegic hand in the BBT, the rmANOVA showed a main effect of Time (*F*_2,14_ = 4.13, *p* = 0.039, p*η*^2^ = 0.37) and a significant tDCS by Time interaction (*F*_2,14_ = 3.76, *p* = 0.049, p*η*^2^ = 0.39), while the main effect of tDCS did not reach significance (*F*_1,7_ = 1.06, *p* = 0.34, p*η*^2^ = 0.13). Post-hoc comparisons showed a significant improvement from baseline only after active tDCS (T0, number of block/min = 18.4 ± 8.1 vs. T1 = 21.9 ± 9.2, *p* = 0.037 and T2 = 21.1 ± 8.1, *p* = 0.049), without difference between the 2 post-tDCS scores (T1 vs. T2, *p* = 0.59). The 3 time points did not differ from each other when sham tDCS was applied (all *p* > 0.6). Importantly, the baseline performance (T0) in the active and sham sessions was comparable (*p* = 0.48) (see Figure 2), excluding possible carry-over practice effects across sessions.

Regarding the nonhemiplegic hand (secondary outcome), no significant effect emerged from rmANOVA: tDCS (*F*_1,7_ = 0.15, *p* = 0.71, p*η*^2^ = 0.02), Time (*F*_2,14_ = 2.72, *p* = 0.1, p*η*^2^ = 0.2), tDCS × Time (*F*_2,14_ = 0.18, *p* = 0.84, p*η*^2^ = 0.03) (see Figure 2).

We further checked for possible carry-over effect induced by receiving active stimulation as first; to this aim we ran a 2-way ANOVA, with the between-subject factor tDCS Order (active first vs. sham first) and the within-subject factor Time (T0 vs. T1): results showed a main effect of Time (*F*_1,6_ = 18.76, *p* = 0.005), confirming significant improvement from anodal stimulation from T0 to T1, but no main effect of tDCS Order (*F*_1,6_ = 0.36, *p* = 0.6), or a significant Time × tDCS Order interaction (*F*_1,6_ = 2.69, *p* = 0.15).

**Figure 3:**
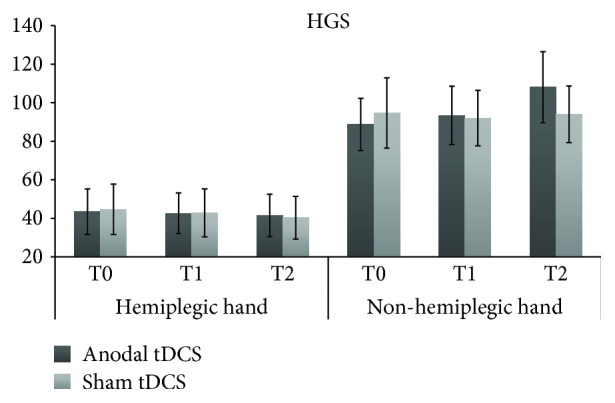
HGS scores (i.e., mean voluntary isometric strength, in kg) for the hemiplegic and nonhemiplegic hands, at each assessment of the real anodal tDCS and sham tDCS sessions. T0 = baseline; T1 = immediately after the end of tDCS; T2 = 90 min after the stimulation session.

HG test could be administered to only six participants, as two subjects did not perform the test due to severe hand impairment. For both hands, rmANOVA did not show any significant effect (see Figure 3): hemiplegic hand, Time (*F*_1,5_ = 0.03, *p* = 0.98, p*η*^2^ = 0.01), tDCS (*F*_2,10_ = 0.76, *p* = 0.5, p*η*^2^ = 0.13), tDCS × Time (*F*_2,10_ = 0.22, *p* = 0.8, p*η*^2^ = 0.04); nonhemiplegic hand, Time (*F*_1,5_ = 0.06, *p* = 0.82, p*η*^2^ = 0.01), tDCS (*F*_2,10_ = 2.56, *p* = 0.1, p*η*^2^ = 0.1), tDCS × Time (*F*_2,10_ = 1.8, *p* = 0.2, p*η*^2^ = 0.2).

### 3.2. Blood Pressure, Heart Rate, and tDCS Side Effects

Blood pressure (mmHg) and heart rate (bpm) were analyzed with the same rmANOVA model used for motor scores; for both, results did not show any changes across time points and between tDCS sessions: heart rate, tDCS (*F*_1,7_ = 0.08, *p* = 0.79, p*η*^2^ = 0.01), Time (*F*_2,14_ = 1.93, *p* = 0.18, p*η*^2^ = 0.03), tDCS × Time (*F*_2,14_ = 0.30, *p* = 0.74, p*η*^2^ = 0.04); blood pressure, tDCS (*F*_1,7_ = 0.05, *p* = 0.83, p*η*^2^ = 0.01), Time (*F*_2,14_ = 0.15, *p* = 0.86, p*η*^2^ = 0.02), tDCS × Time (*F*_2,14_ = 0.97, *p* = 0.40, p*η*^2^ = 0.03).

No participant reported severe adverse effects following stimulation. With respect to the self-report questionnaire assessing tDCS side effects, as shown in Tables 2 and 3, only a limited number of participants reported transient and slight discomfort after stimulation, but this occurred in a similar number of participants, with comparable intensity, during both active (mean number of participants reporting 1 or more side effect = 1.5; mean total score = 0.75) and sham tDCS (mean number of participants reporting 1 or more side effect = 1; mean total score = 1, vs. active tDCS), as assessed by comparing active and sham tDCS with Wilcoxon test: number of participants reporting side effect, *Z* = 0.37, *p* = 0.72, intensity of the reported side effects, *Z* = 1.05, *p* = 0.3.

### 3.3. Exploratory Analysis of Demographic and Lesion Effects

Given the heterogeneity of our small sample with respect to age and lesion size (see Table 1), correlation analyses were performed for BBT, where a significant improvement in tDCS was found. In particular, Pearson correlations were used to test the association between improvement for BBT after active tDCS (T1 *minus* T0) and age (mean age = 17.5 ± 6.1 years) and lesion; the latter considering in different size analyses of hemispheric damage (i.e., mean lesion severity score = 6.8 ± 4.5) and of subcortical damage (i.e., mean lesion severity score = 2.3 ± 1.9), their sum (i.e., mean lesion severity score = 8.9 ± 4.9), and only frontal lobe damage (i.e., mean lesion severity score = 1.8 ± 1.2). All correlation analyses did not show any association between improvement brought about by active anodal tDCS and the considered factor: age (*r* = 0.20, *p* = 0.64), cortical lesion (*r* = −0.40, *p* = 0.38), subcortical lesion (*r* = 0.36, *p* = 0.42), cortical-subcortical lesion (*r* = −0.17, *p* = 0.72), and frontal lobe lesion (*r* = −0.40, *p* = 0.38). To further check for possible effects of age and lesion size, analyses of covariance (ANCOVA) were also performed, with Time (T0 and T1) as within-subject factor and, as linear and interactive covariates, age and abovementioned measures of brain lesion. In every ANCOVA, no significant interaction between Time and covariates was found (all *p* > 0.4).

## 4. Discussion

The main aim of this pilot study was to explore the effects of a single application of anodal tDCS at 1.5 mA (a current intensity so far never tested in UCP) on the motor performance in individuals affected by UCP, considering also the assessment and recording of possible side effects. Results show that a single application of anodal tDCS over the affected M1 can improve, in a safe and well-tolerated way, unilateral manual function (hand dexterity) of subjects with UCP; improvement emerges immediately at the end of stimulation and persists for at least 90 minutes. It is worth noting, improvement was confined to the hemiplegic hand, while performance of the nonhemiplegic one was not influenced by tDCS.

Regarding HGS, no effect was brought about by tDCS. On the one hand, it should be noted that this test was performed on only six participants (see Results), so the absence of the effect could be related to a smaller sample, as compared to BBT. On the other hand, BBT and HGS measure different aspects of motor behavior; the first measures unilateral gross manual dexterity while the second one measures isometric force of voluntary movements. It follows that anodal tDCS may be more useful in changing functional hand performance, closer to real-world object manipulation, rather than lower motor function, such as muscular contractions, at least with the current parameters (intensity, duration, and polarity), and in the case of a single application.

Maintenance of improvement for BBT after 90 minutes is in line with the neurophysiological evidence showing that a single application of anodal tDCS for more than 10 minutes can induce after-effects on motor cortex excitability that last up to 90 minutes [26]. We did not assess whether such motor improvements were maintained over time, although we speculate a return to baseline performance since the two pre-tDCS assessments did not differ from each other and those participants who received active tDCS as first had a T0 score of the sham tDCS session (15.7) almost comparable to that of the active session (T0 = 15). Since stimulation sessions were performed at least 24 hours apart, this indicates that the effect of tDCS was transient, likely disappearing the day after. However, this aspect deserves further empirical investigation.

Finally, our results apparently show no relationship between tDCS effects at BBT and brain lesion timing, site, and severity. Previous studies have demonstrated that timing and severity of brain lesion are related to hand motor function, assessed by function and activity levels, in children with UCP [37, 43]. However, the heterogeneity of our small sample precludes any definitive statement on the absence of the associations between tDCS effects and individual demographic and brain lesion characteristics. Indeed, different plasticity mechanisms are involved in function recovery after unilateral brain lesions according to the involvement of different brain cells and structures. A better understanding of the possible role of brain lesion-related factors to tDCS effects, also through the use of more advanced imaging techniques, is mandatory in order to adapt intervention strategies. We can only speculate that individual patterns of corticospinal reorganization in UCP might impact tDCS efficacy, especially with respect to the hemisphere stimulated and current polarity, more than brain structural abnormalities [26]. Future studies are needed to verify this hypothesis.

Importantly, in line with previous evidence, during this study no serious adverse effects were induced by tDCS both immediately after and in follow-up (90 minutes and 24 hours after tDCS session), providing a first indication on the safety and good tolerance of anodal stimulation at 1.5 mA for 20 minutes in UCP, when NIBS guidelines for safe application are followed [44, 45]. Some side effects occurred in a limited number of participants, but they were mild and transient, and similar in both the active and sham sessions. Moreover, we did not detect any tDCS-related changes in blood pressure, heart rate, rhythm and quality of nocturnal sleep, mood, and daily activities (the latter also checked the day after the stimulation, at 24 hours).

The main limitations of this study are the small size and high heterogeneity of sample. Although to be viewed as preliminary, the evidence from this study supports the potential facilitatory effects of anodal tDCS in promoting improvement of unilateral manual disorders in UCP and suggests the safety of this stimulation approach in the pediatric neurological population.

Further studies on larger samples of subjects with UCP are needed to confirm and broaden our preliminary findings. From a rehabilitation perspective, it will be of interest to combine multiple sessions of anodal tDCS with a motor training, considering that the cathodal tDCS was unable to increase the motor-learning gains in subjects with UCP [26, 30]. The optimal dosage, timing, and montage of tDCS still need to be fully determined, also for adults. Here we provide an initial evidence of the efficacy of a current intensity of 1.5 mA for anodal stimulation; further studies in UCP are required to verify whether the intensity of 1.5 mA could be more, equal, or less effective than other intensities. Moreover, the influences of timing of brain lesion and type of corticospinal reorganization as well as motor and neurological degree of severity need to be further investigated given that our preliminary findings are inclusive in this regard. In this regard, another major limitation of the present study is the absence of the assessment of the neurophysiological status of our UCP participants, which precluded the evaluation of tDCS effects on cortical responses, as well as of the relationship between tDCS-induced behavioral gains and underlying neurophysiology. In the developing brain with neurologic injury, motor outcomes and tDCS effects are both related to differences in the corticospinal circuitry [15, 26, 46]. Finally, the development of specific guidelines for the application of tDCS in the pediatric population could facilitate recruitment and standardization on the use and management of tDCS [44] and potentially lead to a greater role as a therapeutic tool for neurodevelopmental rehabilitation.

## Figures and Tables

**Table 1:** Characteristics of the participants.

No.	tDCS order (a = active, s = sham)	Age	UCP side (R = right, L = left)	UCP form [1]	MACS [34]	MRI global severity score [36, 37]
1	sa	22	L	II	3	na
2	sa	27	R	I	1	15
3	sa	17	L	II	2	9
4	sa	11	L	III	3	4.5
5	sa	10	R	III	2	13.5
6	as	12	L	III	2	14.5
7	as	21	R	II	2	8
8	as	20	R	I	3	6
M ± SD		17.5 ± 6.1	**4**L**: 4R**		2.25 ± 0.70	9.5 ± 3.87

Acronyms: No. = number; M = mean; SD = standard deviation; A = active; UCP = Unilateral Cerebral Palsy; S = sham; R = right; L = left; MACS = Manual Ability Classification System; MRI = magnetic resonance imaging; na = quality of the images not suitable for detailed assessment.

**Table 2:** Adverse effects' questionnaire (early evaluation).

Adverse Effects Questionnaire (AEs-Q) items	Active (*n* = 8 subjects)				Sham (*n* = 8 subjects)			
	No. of subjects reporting tDCS AEs at T1	Mean intensity range of the effect (0 = absent, 1 = mild, 2 = moderate, 3 = severe)	No. of subjects reporting tDCS AEs at T2	Mean intensity range of the effect (0 = absent, 1 = mild, 2 = moderate, 3 = severe)	No. of subjects reporting tDCS AEs at T1	Mean intensity range of the effect (0 = absent, 1 = mild, 2 = moderate, 3 = severe)	No. of subjects reporting tDCS AEs at T2	Mean intensity range of the effect (0 = absent, 1 = mild, 2 = moderate, 3 = severe)
Headache	2	1	2	1.5	2	1.5	2	1
Neck pain	1	1	0	—	1	1.5	1	1
Scalp pain	1	1	0	—	3	1.5	0	—
Burning	2	1	0	—	3	1	0	—
Tingling	3	1	0	—	3	1.5	1	1
Drowsiness	1	0	1	1	1	1	1	1
Lack of concentration	1	0	1	1	0	—	0	—
Feelings change	1	1	0	—	0	—	0	—
Were you afraid?	No				No			
Would you do it again?	8	Yes			8	Yes		

Data represent the number of subjects, both for active and sham tDCS sessions, that reported the specific adverse effects at T1 (immediately after tDCS) and at T2 (90 min after tDCS); if adverse effects were present, the intensity were reported (0 = absent, 1 = mild, 2 = moderate, 3 = severe).

**Table 3:** Side effects' questionnaire at 24 h after tDCS session.

Adverse Effects Questionnaire (AEs-Q) items	Active (*n* = 8 subjects)		Sham (*n* = 8 subjects)	
	No. of subjects reporting tDCS AEs after 24 h (T3)	Mean intensity range of the effect (0 = absent, 1 = mild, 2 = moderate, 3 = severe)	No. of subjects reporting tDCS AEs after 24 h (T3)	Mean intensity range of the effect (0 = absent, 1 = mild, 2 = moderate, 3 = severe)
Difficulty falling asleep	0	—	0	—
Night awakenings	0	—	0	—
Early awakenings	0	—	0	—
Insomnia	0	—	0	—
Daytime sleepiness	1	1	1	1
Reduction of activities	0	—	0	—
Hyperactivity	1	1	1	1
Inattention	2	1	1	1
Irritability	1	1	0	—
Restlessness	1	1	1	1
Sadness	0	—	0	—
Euphoria	0	—	0	—

Data represent the number of subjects, both for active and sham tDCS sessions, that reported the specific adverse effects at T3 i.e., 24 h after the tDCS session; if adverse effects were present, the intensity were reported (0 = absent, 1 = mild, 2 = moderate, 3 = severe). The questionnaire was administrated by telephone.

## Data Availability

The data used to support the findings of this study are included within the article.
